# Therapeutic Patient Education in the Digital Era: Opportunities and Challenges in Diabetes Care

**DOI:** 10.1016/j.mcpdig.2025.100297

**Published:** 2025-10-15

**Authors:** Jorge C. Correia, Katarzyna Wac, Catherine Joly, Jean-Philippe Assal, Surabhi Joshi, Cosette Fakih El Khoury, Zoltan Pataky

**Affiliations:** a Unit of Therapeutic Patient Education, WHO Collaborating Centre, Geneva University Hospitals, Switzerland; b Faculty Diabetes Centre, Faculty of Medicine, University of Geneva, Switzerland; c Quality of Life Technologies Lab, Center for Informatics, University of Geneva, Switzerland; d Fondation recherche et formation pour l'enseignement du malade, Geneva, Switzerland; f Digital Health and Innovation, NCD department, World Health Organization, Geneva, Switzerland; g National Institute of Public Health, Clinical Epidemiology, and Toxicology-Lebanon (INSPECT-LB), Beirut, Lebanon

The prevalence of chronic diseases is steadily increasing worldwide, prompting emphasis on digital health technologies (DHTs) to enhance disease management, improve patient outcomes, and reduce health care costs.^1^ With the rapid technological development, DHTs are transforming health care delivery, including therapeutic patient education (TPE).^2^^,^^3^ Indeed, TPE is widely recognized as the cornerstone for chronic disease management, offering person living with chronic condition(s) (PLwCC) essential tools to understand their condition and their treatment plans and make informed lifestyle changes.^4^ By fostering self-management skills and active participation in care, TPE improves clinical outcomes and quality of life and reduces hospitalizations.^5^, ^6^, ^7^, ^8^ It is crucial to analyze whether the developments of DHTs align with the core principles of TPE, such as personalization, patient autonomy, emotional support, and continuous learning.^4^ Without this alignment, DHTs risk reducing education to simple information transfer.

This commentary discusses opportunities and challenges in integrating DHTs into TPE, using diabetes mellitus as an illustrative case. Diabetes offers a rich context because it is highly prevalent, requires daily self-management, and has a long history of digital innovation.

### TPE: Definition and Core Principles

TPE is a structured, person-centered educational process aimed at equipping PLwCC with the knowledge, skills, and confidence needed for effective self-management.^4^ TPE goes beyond simple knowledge transfer, addressing emotional, psychological, and social dimensions of learning.^9^ The TPE core principles include the following^9^:

• Patient autonomy: capacity to make informed decisions, promoting self-efficacy.
• Personalization: tailored education to patients’ unique needs and contexts.
• Comprehensive approach: considering not only the medical and biophysiological but also the emotional, psychological, and social dimensions of patient care.
• Collaboration: PLwCC collaborate with providers, who serve as facilitators, not authorities.

### Frameworks Underpinning the TPE Model

Therapeutic patient education draws on interdisciplinary frameworks drawn from diverse fields, which enrich the understanding of how patients learn and internalize health-related information (Figure 1)^5^:

• Pedagogy: adult learning theories such as constructivism and experiential learning view learning as active and adaptive, building on prior experience.^10^
• Anthropology: Understanding cultural values helps tailor TPE to the patient’s worldview, enhancing relevance.^11^
• Psychology: Behavioral and cognitive theories highlight self-efficacy and emotions as key to learning readiness and the ability to apply new knowledge.^5^
• Sociology: TPE also leverages social learning theory, which posits that individuals learn by observing the behaviors and outcomes of others, encouraging social learning and motivation.^12^
• Communication and linguistics: Communication theory guides the delivery of clear, accessible, and person-centered TPE.^5^

**Figure 1:**
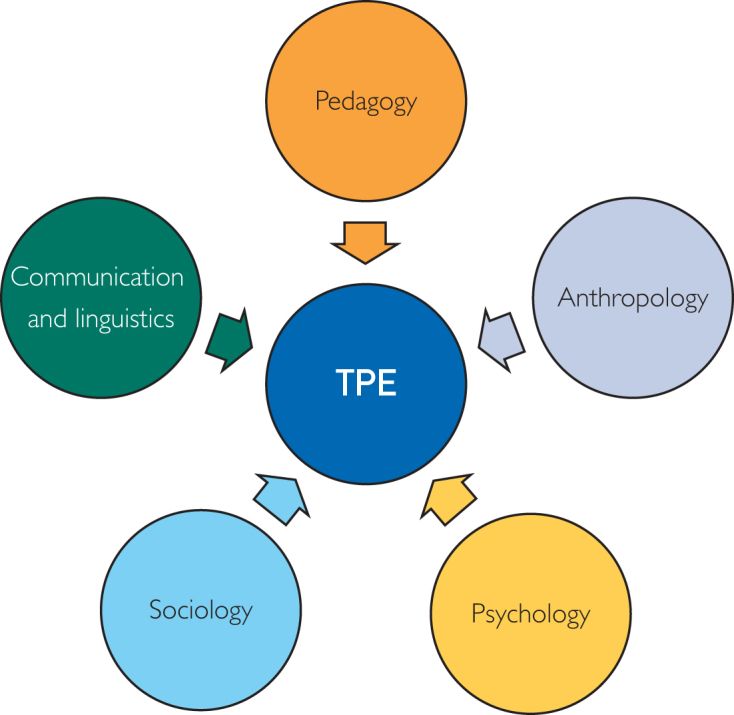
Frameworks underpinning the TPE model. TPE, therapeutic patient education.

These interdisciplinary insights ensure that TPE is not only informative but also culturally relevant, behaviorally grounded, and emotionally supportive.

### Potential of Digital Health Technologies in TPE

Digital health technologies encompass a range of tools and systems that leverage digital innovations, such as mobile health applications, wearable devices, artificial intelligence (AI), telemedicine platforms, and virtual or augmented reality, to improve health care delivery and patient outcomes. These technologies should be complementary to TPE as described further.

#### Personalized and Continuous Engagement

TPE emphasizes tailoring educational interventions to the learner’s needs, and DHTs can support this through their current developments and integration in personal services, such as data analytics and AI, digital platforms that can adjust educational content in real-time, aligning with the patient’s existing knowledge and learning pace.^13^

For example, applications such as mySugr and Glooko integrate glucose, nutrition, and activity data, offering personalized coaching and educational modules.^14^^,^^15^ Continuous glucose monitoring (CGM) systems, empower patients to track glycemic trends continuously and learn how diet, exercise, and medication affect glucose control.^16^

#### Engagement Through Interactive and Immersive Tools

DHTs offer various interactive and immersive tools, such as virtual reality simulations and interactive online modules, which can significantly enhance patient engagement.^3^^,^^17^^,^^18^ These tools allow patients to explore and manipulate information actively, which is crucial for reshaping their mental models. For example, virtual reality simulations are being piloted in diabetes care to let patients visualize how glucose levels respond to food intake, activity, or insulin administration.^19^

#### Continuous Learning Opportunities

Learning is a continuous process, not confined to specific moments of instruction. DHTs support this by providing PLwCC with ongoing access to educational resources. Whether through telemedicine platforms, wearable devices, or online educational resources, PLwCC can continuously engage with their education, reinforcing and expanding their knowledge over time.^2^

For instance, applications linked to CGM systems provide continuous access to personalized feedback. People living with diabetes can revisit their glucose trends, dietary choices, and insulin patterns at any time, reinforcing lessons from structured TPE sessions and maintaining adherence as their condition evolves.^20^

#### Monitoring Trends and Simulating Future Health Outcomes

DHTs such as application interfaces that monitor trends or use digital twins can simulate future health trajectories based on past behaviors.^3^^,^^21^ In diabetes care, digital twins are being tested to model the impact of lifestyle changes or insulin adjustments on long-term HbA1c control, cardiovascular disease risk, and complication rates. These simulations give patients personalized insights into how their current habits might influence future outcomes, helping them visualize the long-term effects of daily choices such as physical activity or carbohydrate intake.^22^^,^^23^

In addition, AI-driven tools are advancing diabetes management by transforming raw data into actionable recommendations. For example, DreaMed Advisor Pro uses CGM and insulin dosing data to recommend personalized insulin adjustments.^24^ Predictive algorithms are also being developed to anticipate episodes of hypoglycemia or hyperglycemia, enabling proactive adjustments. Such applications demonstrate AI’s capacity to transform education into proactive, personalized support.

#### Motivations and Emotional States

DHTs enhance motivation through digital coaches that deliver timely prompts based on patient progress and emotional state, reinforcing concordance to care plans. For example, platforms like BlueStar offer feedback that reframes setbacks (eg, glucose variability) into opportunities for learning and improvement, reducing discouragement.^25^

Gamification and adaptive learning strategies also reinforce motivation by making education more engaging. Applications such as BlueStar integrate behavioral nudges, goal tracking, and tailored educational content to encourage healthy habits. Online communities, such as PatientsLikeMe and diabetes-specific forums, provide opportunities for peer learning and collaborative problem solving.

Instead of focusing solely on fluctuating clinical outcomes, digital tools can highlight effort-based achievements such as adherence to glucose logging, consistent physical activity, or persistence with dietary tracking. For example, mySugr rewards patients with playful feedback when they log meals or blood glucose readings, reinforcing the value of consistency.^14^ DHTs emphasize effort and perseverance, reinforcing TPE’s aim of reshaping self-efficacy and mental frameworks and sustaining behavior change.

#### Social Environment

TPE highlights the importance of peer learning, shared experiences, and collaborative support in fostering patient autonomy and adherence to self-management strategies. DHTs can amplify this aspect by creating platforms for moderated and meaningful social engagement.^26^

In diabetes, this is seen in peer-support forums linked to applications such as Glooko^15^ or in broader communities like PatientsLikeMe,^27^^,^^28^ where people managing diabetes exchange data, discuss challenges, and celebrate successes.^27^^,^^28^

For instance, people living with diabetes managing their condition might share wearable glucose monitor data within a group of peers also using the same technology. Observing others successfully managing similar health challenges can inspire confidence, provide practical tips, and create accountability through communal support.

Health care professionals (HCPs) also play a central role in diabetes management, particularly for children, adolescents, and older adults. DHTs such as Dexcom Share enable HCPs to remotely monitor glucose data and receive alerts for hypoglycemia or hyperglycemia, providing reassurance and supporting timely intervention. Telehealth systems increasingly include caregiver education modules on insulin administration, carbohydrate counting, and recognizing early signs of complications. By actively involving HCPs, DHTs extend the reach of TPE from the patient to the family and community context.

Although social comparison can be a powerful motivator, however, its impact is not universally positive. For some individuals, comparing their progress to others who may be further along in their journey can lead to feelings of inadequacy or frustration. Therefore, the presence and design of this feature must be personalized to every PLwCC. A comparative overview of commonly used digital health tools in diabetes care and their clinical utility is presented in Table, which summarizes how different categories of DHTs contribute to TPE by supporting self-management, caregiver engagement, and personalized feedback.

**Table:** Common DHTs and Their Utility in Diabetes Management

DHT type	Example	Key features	Clinical utility in diabetes
Mobile applications	mySugr, Glooko, BlueStar	Symptom and behavior tracking, personalized education, and coaching	Improves adherence and daily self-management
Continuous glucose monitoring	Dexcom, FreeStyle Libre	Real-time glucose trends, alerts, and data sharing	Enhances TPE, reduces hypoglycemia/hyperglycemia, supports caregiver involvement
Wearables	Fitbit, Apple Watch	Activity and lifestyle tracking	Encourages physical activity and behavioral change
AI-driven Platforms	DreaMed Advisor Pro, Digital Twin models	Insulin adjustment support and predictive analytics	Provides personalized feedback and proactive care
Caregiver Platforms	Dexcom Share, telehealth modules	Shared dashboards, alerts, and caregiver education	Strengthens caregiver role in diabetes management

AI, artificial intelligence; DHT, digital health technology; TPE, therapeutic patient education.

### Limitations of DHTs in TPE

#### Limited Adaptability and Context Sensitivity

Effective TPE requires tools to adapt not just to static user inputs but also to the dynamic interplay of a patient’s physical, emotional, and psychological states.^29^^,^^30^

For example, people living with diabetes experiencing anxiety about glucose fluctuations may disengage from an application that focuses solely on numerical goals without providing emotional support or stress management strategies. Tools that incorporate emotional intelligence (eg, AI systems capable of detecting stress via voice or biometric feedback) could better align with TPE’s holistic approach, supporting both emotional well-being and cognitive learning processes.

#### Overreliance on Information Delivery

DHTs often prioritize information dissemination over fostering deeper, reflective learning. Therapeutic patient education stresses that effective learning involves the transformation and integration of new knowledge into existing mental frameworks.^31^

For example, a diabetes education application that only lists dietary guidelines may be less effective than one offering interactive, scenario-based learning—such as planning a balanced meal and receiving real-time feedback on its carbohydrate content and projected glucose impact.

#### Digital Literacy and Socioeconomic Barriers

Although DHTs have democratized access to health care information, they remain inaccessible to many owing to disparities in digital literacy and socioeconomic resources, challenges that are amplified in low- and middle-income countries.^30^^,^^32^ People living with diabetes with limited technical skills may find it challenging to navigate complex interfaces, while those in underserved regions may lack reliable internet access or the resources to afford CGM or smart devices.^30^^,^^32^

Older adults with type 2 diabetes, for instance, may hesitate to use applications that require multiple steps for glucose entry or insulin logging. This limits the reach of DHTs and risks widening health disparities unless tools are designed with simplicity, accessibility, and affordability in mind.

#### Data Privacy and Trust Issues

Patients’ trust in DHTs is pivotal for engagement and learning, but concerns about data privacy and security undermine confidence. When patients are uncertain about how their health data are stored or shared, they may disengage entirely, reducing the potential impact of TPE.^2^^,^^3^^,^^30^^,^^31^ For example, a CGM platform that transmits glucose data to the cloud without transparent policies on storage or third-party access may lead patients to disable sharing functions.

#### Perceived or Poor Accuracy of DHTs

A significant limitation of many DHTs is the perceived or actual inaccuracy of the information and data they provide. Patients may doubt the precision of health applications, wearable devices, or AI-driven recommendations, which can undermine trust and engagement.^29^^,^^30^ If DHTs deliver inaccurate health metrics or offer generic advice that does not align with a patient’s experience, it may weaken the learning process. In diabetes, CGM systems occasionally produce discrepancies owing to calibration errors or sensor delays, while insulin dose calculators may generate generic advice not tailored to individual patterns.

#### Overwhelming Amount of Data

Another challenge is the sheer volume of data that many DHTs generate. Although access to real-time metrics can be empowering, it can also overwhelm patients, particularly those who lack strong digital literacy.^30^ Constant streams of information can make it difficult for patients to focus on actionable insights, reducing the effectiveness of education. The allosteric model suggests that learning should be focused and manageable; too much information can hinder a patient’s ability to reflect and adapt effectively.

For example, people with diabetes may feel confused when confronted with multiple metrics (glucose variability, time-in-range, carbohydrate ratios, and activity levels) without guidance on which require immediate action. To be effective in TPE, educational components must translate raw data into clear, actionable insights, helping patients focus on priorities such as avoiding hypoglycemia or improving time-in-range rather than being overloaded with graphs and numbers.

#### Limited Integration with Health Care Systems

Many DHTs operate independently of the broader health care ecosystem, limiting their effectiveness in TPE. For patients to effectively integrate new knowledge and skills, their learning experience should align with clinical care and provider feedback. Digital health technologies that fail to integrate with electronic health records or lack communication channels with health care professionals may lead to fragmented care, reducing the impact of patient education on long-term health outcomes.^30^

For instance, a patient may diligently track glucose in an application, but if the data cannot be integrated into the clinician’s electronic health record, opportunities for personalized feedback during consultations are missed.

#### Behavioral Overload and User Fatigue

Although DHTs encourage continuous engagement, they can also contribute to user fatigue. Patients may become overwhelmed by constant notifications, reminders, or educational prompts, feeling judged and inadequate in self-management efforts, which may lead to disengagement. Behavioral overload can diminish motivation and create frustration, particularly if patients feel they cannot keep up with their digital interventions.^30^

For example, a diabetes application that repeatedly notifies users of out-of-range glucose values without offering practical coping strategies may demotivate rather than support them. By contrast, TPE-informed design would balance alerts with positive reinforcement, celebrating effort-based achievements (eg, consistent logging or improved carb counting) even if glucose outcomes remain variable. Opportunities and challenges of DHTs for TPE are illustrated in Figure 2.

**Figure 2:**
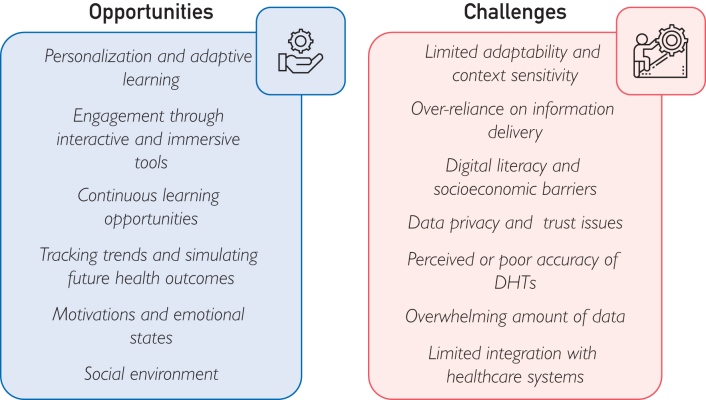
Opportunities and challenges of DHTs for TPE. DHT, digital health technology; TPE, therapeutic patient education.

## Discussion

DHTs hold strong potential for enhancing TPE by providing personalized, engaging, and continuous learning opportunities that reflect its adaptive principles. Yet, the empathy, trust, and contextual sensitivity of in-person consultations remain irreplaceable in chronic disease care. A hybrid model that combines the scalability of DHTs with the human depth of patient-provider relationships offers the most effective path forward.

In-person interactions foster dialogue, emotional support, and trust; elements essential for addressing sensitive issues such as lifestyle change or mental health. Providers can also interpret nonverbal cues (eg, body language, tone, and facial expression) that reveal hidden concerns and allow real-time adjustments, capacities beyond digital tools.

To maximize the potential of both digital and in-person modalities, the following strategies are suggested:

• Hybrid education models: Combine DHT scalability with in-person sessions and workshops, for example, applocations for daily monitoring and periodic group discussions.
• Leverage DHTs to enhance in-person consultations: Use DHTs to collect data between visits, enabling more personalized, insight-driven interactions.
• Training for HCPs in digital integration: Equip providers to interpret digital data and integrate it into TPE, ensuring technology complements human care.

## Conclusion

The future of TPE lies in the seamless integration of digital innovation and human connection. Although DHTs bring scalability, precision, and personalization, the irreplaceable aspects of human interaction—empathy, trust, and context sensitivity—provide the emotional and experiential foundation for transformative learning. By combining these strengths, health care systems can deliver more effective, TPE that empowers individuals to manage their chronic conditions with confidence and resilience.

## Potential Competing Interests

The authors report no competing interests.
